# Mediators of Racial Inequities in Non‐Small Cell Lung Cancer Care

**DOI:** 10.1002/cam4.70757

**Published:** 2025-03-07

**Authors:** Safraz A. Hamid, Do H. Lee, Jeph Herrin, James B. Yu, Craig E. Pollack, Lorraine T. Dean, Jacquelyne J. Gaddy, Carol R. Oladele, Shelli L. Feder, Maureen E. Canavan, Marcella Nunez‐Smith, Pamela R. Soulos, Cary P. Gross

**Affiliations:** ^1^ Department of Surgery Yale School of Medicine New Haven Connecticut USA; ^2^ Yale National Clinician Scholars Program New Haven Connecticut USA; ^3^ Cancer Outcomes, Public Policy and Effectiveness Research (COPPER) Center, Yale Cancer Center New Haven Connecticut USA; ^4^ Department of Internal Medicine Yale School of Medicine New Haven Connecticut USA; ^5^ Department of Radiation Oncology St. Francis Hospital and Trinity Health of New England Hartford Connecticut USA; ^6^ Department of Health Policy and Management Johns Hopkins Bloomberg School of Public Health Baltimore Maryland USA; ^7^ John Hopkins School of Nursing Baltimore Maryland USA; ^8^ Department of Epidemiology Johns Hopkins Bloomberg School of Public Health Baltimore Maryland USA; ^9^ Equity Research and Innovation Center (ERIC) Yale School of Medicine, Yale University New Haven Connecticut USA; ^10^ Yale School of Nursing Orange Connecticut USA; ^11^ VA Connecticut Healthcare System West Haven Connecticut USA

**Keywords:** mediation analysis, non‐small cell lung cancer, quality of care, racial inequities, structural racism

## Abstract

**Background:**

Black patients with non‐small cell lung cancer (NSCLC) are more often diagnosed at a later stage and receive inadequate evaluation and treatment compared to White patients. We aimed to identify factors representing exposure to structural racism that mediate the association between race and NSCLC care.

**Methods:**

We queried Surveillance, Epidemiology, and End Results–Medicare for non‐Hispanic Black and White patients ≥ 67 years diagnosed with NSCLC from 2013 to 2019. Our outcomes were localized diagnosis stage, receipt of stage‐appropriate evaluation, receipt of stage‐appropriate treatment, two‐year survival, and receipt of “optimal” care, an aggregate metric comprising the first three listed outcomes. We estimated indirect effects of mediators on the association between race and outcomes.

**Results:**

Of 69,130 patients, 8.2% were Black. Medicare–Medicaid dual eligibility, a marker of individual‐level socioeconomic status (SES), accounted for the largest proportion of mediating effects for most outcomes, ranging from 13.6% (*p* < 0.001) for localized diagnosis stage to 25.0% (*p* < 0.001) for two‐year survival. Receipt of an influenza vaccine, a marker of health care access, had the second largest mediating effects on the associations between race and diagnosis stage (9.5%, *p* < 0.001), treatment (15.3%, *p* < 0.001), and optimal care (11.4%, *p* < 0.001). Neighborhood‐level SES accounted for the third largest proportion of the effects of race on each outcome, explaining between 9% and 16% of the racial inequities at each phase (all *p* < 0.001).

**Conclusions:**

Individual‐ and neighborhood‐level structural factors partly explain inequities in NSCLC care, and their effects vary based on the phase of care. Interventions should be adapted to the phase of care.

## Graphical abstract

Individual‐ and neighborhood‐level factors mediate the observed racial inequities in the quality of non‐small cell lung cancer (NSCLC) care, and their effects vary based on the phase of cancer care. The design of interventions addressing these structural drivers of inequitable care should be tailored based on the phase of NSCLC care.

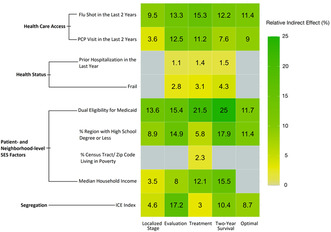

## 1 Introduction

Non‐small cell lung cancer (NSCLC), the leading cause of cancer death, exerts a disproportionate burden across subgroups in the United States. In particular, racial inequities exist along the spectrum of care, from diagnosis and evaluation to treatment and survival. Black patients are less likely than White patients to be diagnosed at an early stage [1] and to undergo guideline‐recommended PET and CT imaging [2]. Black patients are also less likely than White patients to undergo surgery [3] and, when they do, they are less likely to receive appropriate lymph node examination [4, 5]. In aggregate, these multiple lapses in care contribute to substantially higher mortality for Black patients [6].

These inequities stem from a society where historical and contemporary structural racism perpetuates the marginalization of Black people in nearly all aspects of life [7, 8]. Structural racism refers to the “totality of ways in which societies foster racial discrimination through mutually reinforcing [inequitable] systems of housing, education, employment, earnings…and health care” [7, 8]. According to the socioecological model of health, exposure to structural racism occurs at multiple levels, from the individual and interpersonal levels to the community and policy levels [9, 10]. For example, at the individual level, Black people experience greater comorbidity burden [11, 12] and are more frequently hospitalized [13] compared to White people. At the community level, the neighborhoods Black people reside are often the most economically and racially segregated [14] and, at the policy level, Black people experience discriminatory housing systems driven by inequitable public and private lending laws [15, 16].

Exposure to structural racism is hypothesized to explain the association between race and health and, through mediation analysis, the contribution of structural racism to racial inequities in cancer care has become clear [17, 18, 19, 20, 21]. Studies have highlighted the mediating effects of health care access at the diagnosis phase of cervical cancer [17], individual‐level socioeconomic status (SES) at the evaluation phase of prostate cancer [18], and neighborhood‐level education at the treatment phase of breast cancer [21]. In the case of NSCLC, studies focusing on a singular phase of care, such as the evaluation phase [22] and the treatment phase [23], have demonstrated that these and other factors related to structural racism are associated with low quality of care among the general NSCLC patient population. For instance, low individual‐ [24] and area‐level [25] SES are both associated with advanced diagnosis stage, while poor health care access has been shown to be associated with mortality [26]. Little is known, however, whether these factors account for the observed racial inequities in NSCLC care.

The design of interventions to promote equity along the continuum of NSCLC care can be informed by understanding which factors are the greatest contributors to racial inequities and whether these vary based on the phase of care. Accordingly, using mediation analysis, we aimed to characterize the relative contribution of factors that mediate the association between race and quality of care at each phase of NSCLC care. Importantly, we sought to investigate factors that are markers of exposure to structural racism along various levels of the socioecological model of health. Through this analysis, we hope to inform phase‐specific levers of change along the continuum of NSCLC care.

## 2 Materials and Methods

### 2.1 Data Source and Study Sample

**FIGURE 1:**
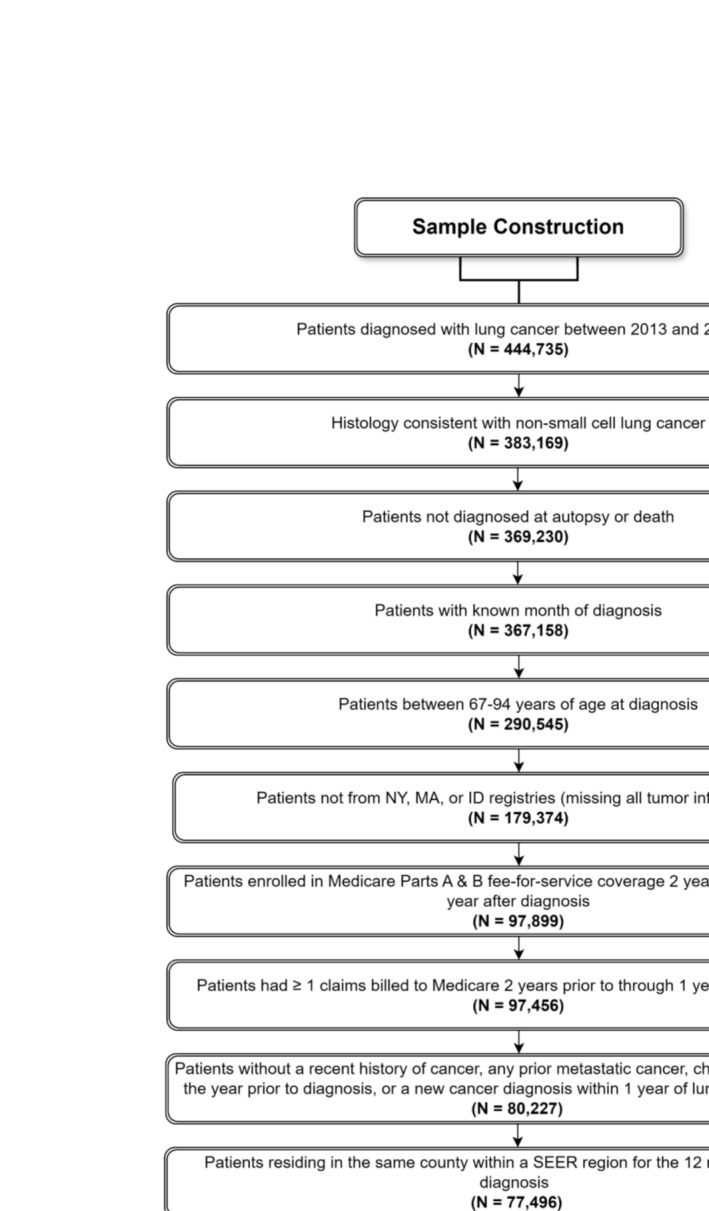
Sample construction.

We used the Surveillance, Epidemiology, and End Results (SEER)‐Medicare database to perform a retrospective cohort study. This database is the linkage of two population‐based data sources; the SEER program of cancer registries collects clinical, demographic, and cause of death information for persons with cancer, and Medicare claims include health care services from the time of a person's Medicare eligibility until death. We included patients ≥ 67 years who were diagnosed with NSCLC from 2013 to 2019 (Figure 1). Eligible patients had continuous fee‐for‐service and Part B coverage from 24 months before through 12 months after cancer diagnosis or death if they died before 12 months. Due to our focus on Black health, we limited our sample to patients who were identified as either non‐Hispanic Black or non‐Hispanic White. The Yale Human Investigation Committee determined that this study did not constitute human participants research. We reported our mediation analysis in accordance with the consensus‐based Guideline for Reporting Mediation Analyses (AGReMA) [27].

### 2.2 Study Variables

#### 2.2.1 Independent Variable

Our independent variable was race, categorized as non‐Hispanic Black or non‐Hispanic White. We used the Medicare Research Triangle Institute (RTI) race variable, which has been validated against patient self‐reported race/ethnicity data [28].

#### 2.2.2 Outcome Measures

Our outcomes were disease diagnosed at a localized stage, receipt of stage‐appropriate evaluation, receipt of stage‐appropriate treatment, and two‐year survival. Localized stages of diagnosis were defined as stages I–IIIA/N1. Stage‐appropriate evaluation and treatment were defined according to National Comprehensive Cancer Network (NCCN) guidelines [29] (Tables S1 and S2). Additionally, we assessed overall quality of care with an “optimal” care metric. This was a binary “yes/no” variable defined as having a localized stage of diagnosis along with receipt of both stage‐appropriate evaluation and stage‐appropriate treatment. Two‐year survival was a binary (yes/no) variable indicating whether a patient survived for 2 years after diagnosis.

#### 2.2.3 Candidate Mediators

**FIGURE 2:**
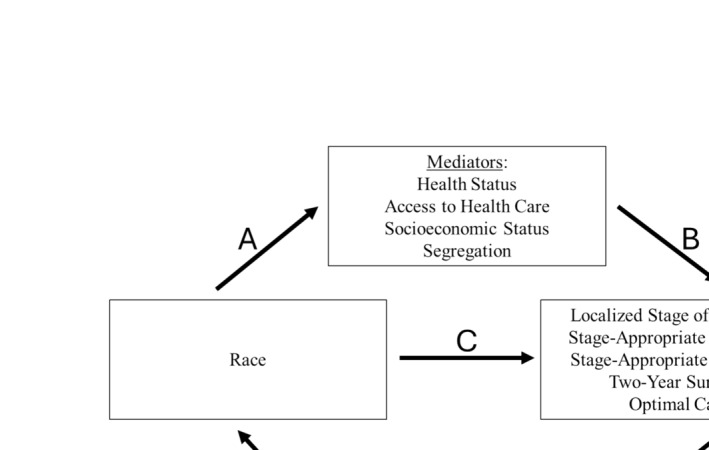
Conceptual model for the effect of race on outcomes. The total effect of race on the five binary outcomes comprises the direct effect (C) and the indirect effects of the mediators (the product of A and B). Age, sex, marital status, and year of diagnosis are conceptualized as confounders in the model.

A mediation analysis assumes a direct causal effect between the exposure (race) and the outcome (quality of care). Additional variables, the mediators, can intervene on this pathway such that the total effect of the exposure on the outcome is, to some degree, explained by the mediators. We considered four categories of mediators that spanned multiple levels of the socioecological model of health and, collectively, represented exposure to structural racism: health status, access to healthcare, SES, and racial and economic segregation (Figure 2). These included both patient‐ and neighborhood‐level variables. Variables that were markers of health status included Elixhauser comorbidity count based on the 24 through 3 months prior to diagnosis, a record of a hospital admission in the year prior to diagnosis, and claims‐based frailty index (CFI) in the year prior to diagnosis. As markers of access to healthcare, we examined receipt of an influenza vaccine and a primary care physician (PCP) visit in the 2 years prior to diagnosis.

Our patient‐level marker of SES was Medicare‐Medicaid dual eligibility, a known predictor of many health‐related processes and outcomes [30, 31, 32]. We considered a patient to be dual eligible if they had at least 1 month of dual eligibility in the 12 months prior to cancer diagnosis. For neighborhood‐level SES factors, we examined the effect of living in a census tract or ZIP code where 20% or more of residents live below the poverty level, a value above which an area is considered to be exhibiting persistent poverty [33]. Additionally, we investigated the effect of a census tract or ZIP code's proportion of patients with a high school degree or less.

Our measure of racial and economic segregation was the Index of Concentration at the Extremes (ICE). This index quantifies the extent to which an area's population is concentrated into extremes of racialized economic privilege or disadvantage [34, 35, 36]. Using 5‐year data (2015–2019) from the US Census American Community Survey, we divided the difference between the number of privileged (80th percentile of US household income, non‐Hispanic White race) and disadvantaged (20th percentile of US household income, non‐Hispanic Black race) individuals in a county by the total Black and White population. We categorized these values into quintiles, with the highest (fifth) quintile representing the most privileged counties.

### 2.3 Statistical Analysis

We described the distribution of sociodemographic, clinical, and socioeconomic characteristics of the sample by race using tests of standard mean differences (SMD). SMD values of 0.2 approximate a small effect size, 0.5 a medium effect size, and 0.8 a large effect size. We assessed the intra‐county correlation coefficient for each outcome; since all were less than 5%, we did not account for autocorrelation in our models [37]. We used chi‐square and *t*‐tests to examine the association between race and each of our outcomes. We then constructed multivariable logistic regression models to evaluate the association between race and each outcome, adjusting for age, sex, and year of diagnosis.

We performed multiple mediation analyses to quantify the extent to which racial inequities in the quality of NSCLC care are mediated by racial differences in our candidate mediators. In our mediation analysis, we conceptualized the effect of race as being mediated by the association between race and candidate mediators, which indirectly contribute to racial inequities in the quality of NSCLC care (Figure 2). Importantly, we hypothesized that the relative strength of these mediators in mediating racial inequities would differ based on the phase of care (Figure S1).

To perform this analysis, we built five models, one for each of our outcomes. In each model, race was included as the independent variable (exposure). We then assessed which variables among our four categories of candidate mediators satisfied criteria to be a potential mediator. To be included as a mediator, a variable had to be significantly associated (*p* < 0.05) with both race and the outcome in bivariate analysis. Those variables not conceptualized as candidate mediators (i.e., not on the causal pathway from race to outcome) but that were significantly associated (*p* < 0.05) with both race and the outcome were treated as confounders and retained in the corresponding model. These included patient age, sex, marital status, and diagnosis year. Variables that we found to be associated with the outcome but not with race were kept in the corresponding model as covariates. We assessed collinearity among the variables in our models by using the variance inflation factor [38]. For our mediation model assessing two‐year survival, we expected earlier phases of care (i.e., diagnosis stage, evaluation, and treatment) to mediate racial inequity in survival. However, we did not include these factors as mediators because we wanted to highlight the mediating effects of upstream factors that were related to exposure to structural racism and that could be targeted to mitigate inequity. Assumptions of our conceptualized mediation models are the presence of no unmeasured confounders for the exposure‐outcome and the mediator‐outcome relationships and no omitted mediation effects.

For each model, we estimated the total effect of race, the direct effect of race, and the indirect effect of each mediator. These effects were computed using the product‐of‐coefficients approach as described by Baron and Kenny [39]. We defined the indirect effect (i.e., the mediated effect) as the portion of the race‐outcome association that was explained by the mediating variables. In our study, this can be interpreted as how the quality of NSCLC care for Black patients would improve if the distributions of potential mediators among Black patients were equivalent to those of White patients. The direct effect is defined as the portion of the race‐outcome association not attributable to the examined mediators. In our study, the direct effect can be interpreted as racial inequity that does not occur through our selected mediators. The total effect is the sum of the direct and indirect effects. To facilitate comparisons of the strength of mediation, we report the relative indirect effects of each mediator, which we defined as the ratio of the indirect effect to the total effect. This ratio reflects the proportion of the effect of race on the outcome that is mediated by the identified variables.

We used 500 bootstrap resampling iterations to estimate standard errors for the indirect effects. We performed most analyses using Stata, version 18.0 (StataCorp, College Station, TX); mediation analyses were performed using the mma software package in R version 4.3.1 (R Group for Statistical Computing).

## 3 Results

We identified 69,130 patients, 5679 (8.2%) of whom were Black. The mean [SD] age was 77.6 [6.6] years, and 50.5% were female. Black patients were younger than White patients and were less likely to visit a PCP (72.2% vs. 80.7%, SMD = 0.20) and receive an influenza vaccine (52.0% vs. 70.0%, SMD = 0.38) in the last 2 years (Table 1). Additionally, Black patients were more likely to be Medicare‐Medicaid dual eligible (37.3% vs. 13.6%, SMD = 0.57) and live in the most economically and racially segregated regions (43.5% vs. 12.4%, SMD = 0.79).

**TABLE 1:** Sample characteristics by patient race.

	Black (*N* = 5679)	White (*N* = 63,451)	SMD^a^
	*N* (%)	*N* (%)	
Sociodemographic factors			
Age (Years)			
67–69	979 (17.2)	8175 (12.9)	0.19
70–74	1656 (29.2)	16,693 (26.3)	
75–79	1427 (25.1)	15,891 (25.0)	
80–84	958 (16.9)	12,388 (19.5)	
85–94	659 (11.6)	10,304 (16.2)	
Female	2729 (48.1)	32,178 (50.7)	0.05
Married	1852 (32.6)	30,967 (48.8)	0.34
Health care access			
Flu shot in the last 2 Years	2952 (52.0)	44,404 (70.0)	0.38
PCP visit in the last 2 Years	4105 (72.3)	51,203 (80.7)	0.2
Health status			
Elixhauser Comorbidity Conditions			
0	1404 (24.7)	15,631 (24.6)	0.14
1–2	1811 (31.9)	23,955 (37.8)	
≥ 3	2464 (43.4)	23,865 (37.6)	
Had ≥ 1 Hospitalization(s) in the Last Year	1685 (29.7)	16,485 (26.0)	0.08
Frail	3280 (57.8)	39,415 (62.1)	0.09
Patient‐ and neighborhood‐level SES factors			
Dual Eligibility for Medicaid	2118 (37.3)	8631 (13.6)	0.57
% of region with high school degree or less			
< 30	685 (12.1)	17,880 (28.2)	0.58
30 to < 40	697 (12.3)	11,956 (18.8)	
40 to < 50	1001 (17.6)	12,608 (19.9)	
50 to < 60	1305 (23.0)	10,382 (16.4)	
60–100	1962 (34.5)	10,404 (16.4)	
Missing	29 (0.5)	221 (0.3)	
% of region living in poverty			
< 5	373 (6.6)	13,376 (21.1)	0.87
5 to < 10	670 (11.8)	17,541 (27.6)	
10 to < 20	1478 (26.0)	20,099 (31.7)	
20–100	3129 (55.1)	12,214 (19.2)	
Missing	29 (0.5)	221 (0.3)	
Median household income^b^	44,100 (22,800)	63,300 (29,200)	0.74
Segregation			
ICE index quintile			
First (most disadvantaged)	> 2459 (> 43.3)	7846 (12.4)	0.79
Second	714 (12.6)	7748 (12.2)	
Third	135 (2.4)	4550 (7.2)	
Fourth	290 (5.1)	7766 (12.2)	
Fifth (most privileged)	2070 (36.5)	35,527 (56.0)	
Missing	< 11 (< 0.2)	14 (0.0)	

Abbreviations: ICE, Index of Concentration at the Extremes; PCP, primary care physician; SES, socioeconomic status.

^a^ Standardized Mean Difference (SMD) is used to report the distribution of the independent variables by race.

^b^ Mean and standard deviations are calculated for these independent variable(s).

**FIGURE 3:**
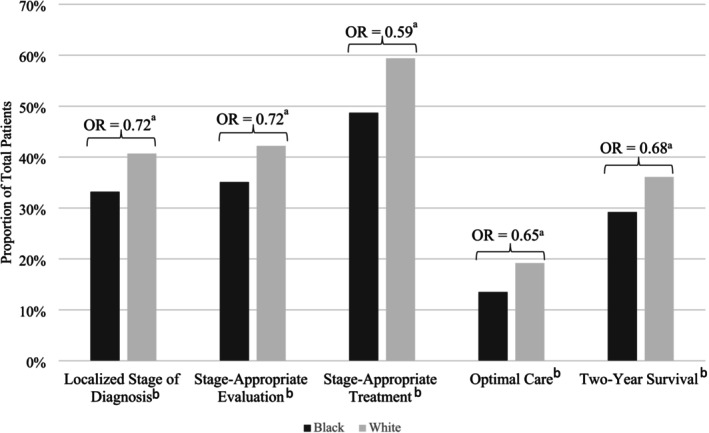
Association between race and each outcome. ^a^Statistically significant multivariate association between race and outcome after adjusting for sex, age, and year of diagnosis. ^b^Statistically significant bivariate association between race and outcome (Chi‐square Test).

In unadjusted analysis, Black patients were less likely than White patients to be diagnosed at a localized stage (33.2% vs. 40.7%), receive stage‐appropriate evaluation (35.1% vs. 42.2%), receive stage‐appropriate treatment (48.7% vs. 59.4%), receive optimal care (13.5% vs. 19.2%), and survive at least 2 years after diagnosis (29.2% vs. 36.1) (all *p* < 0.001) (Figure 3). After adjusting for age, sex, and year of diagnosis, these inequities persisted. Black patients had lower odds of being diagnosed at an early stage (odds ratio (OR) 0.72, 95% confidence interval (CI): 0.68–0.76), receiving stage‐appropriate evaluation (OR 0.73, 95% CI: 0.68–0.77), receiving stage‐appropriate treatment (OR 0.59, 95% CI: 0.56–0.63), receiving optimal care (OR 0.65, 95% CI: 0.60–0.71), and surviving at least 2 years after diagnosis (OR 0.69, 95% CI: 0.65–0.73).

Variables that met criteria for mediators included prior influenza vaccine, prior hospitalization, prior PCP visit, frailty, dual eligibility for Medicare‐Medicaid, percentage of census tract/zip code with high school degree or less, percentage of census tract/zip code below poverty level, median household income, and the ICE. Elixhauser comorbidity count did not meet criteria since it was not associated with race. However, because it was associated with localized diagnosis stage, stage‐appropriate evaluation, two‐year survival, and optimal care, it was included as a covariate in these models.

**FIGURE 4:**
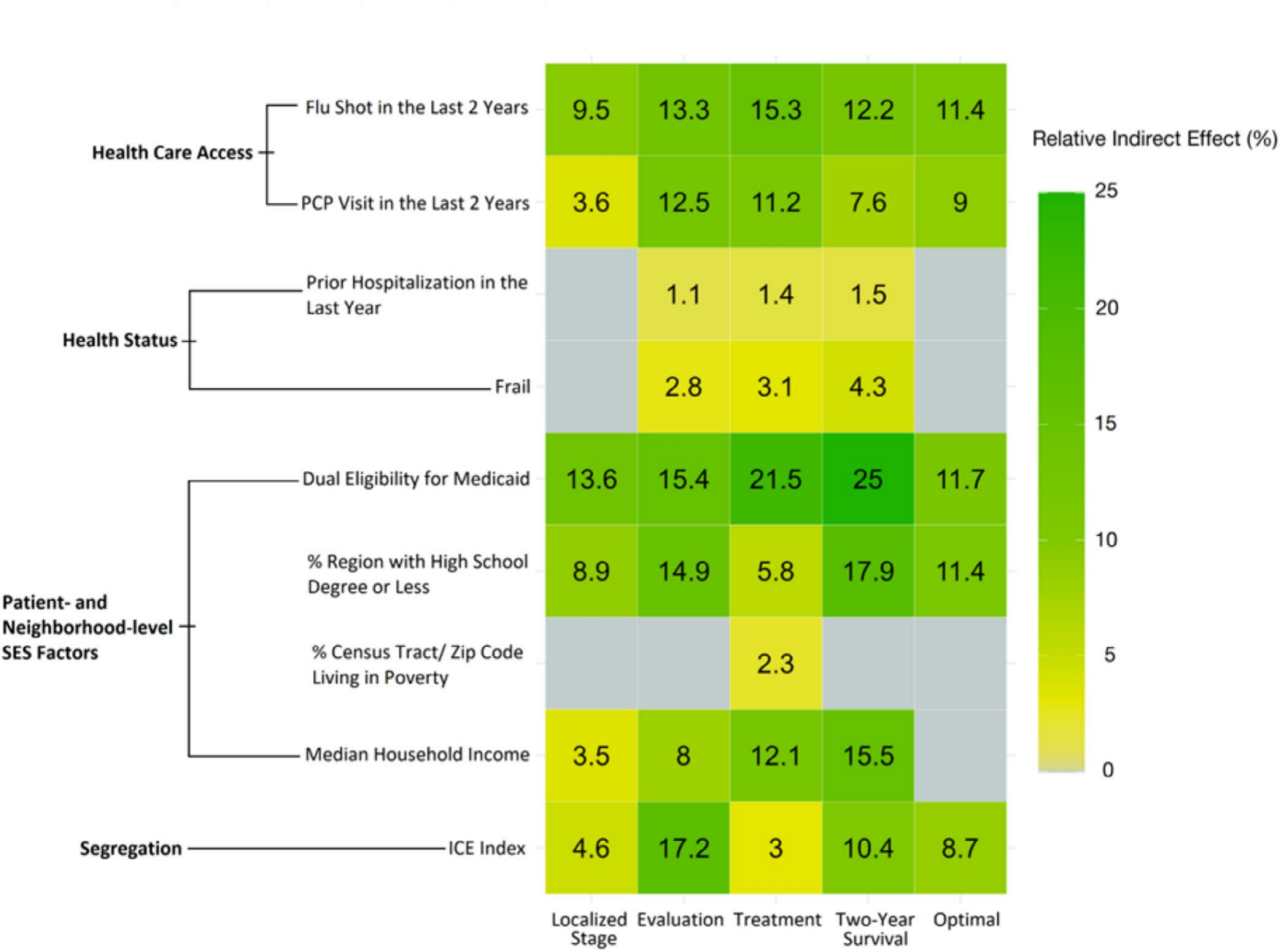
Heat map of relative indirect effects of mediators. Darker tiles (green) indicate stronger relative indirect effects; lighter tiles (yellow) indicate weaker relative indirect effects. Gray tiles indicate no statistically significant mediation or inconsistent mediation. Inconsistent mediation occurs when the sign of the indirect effect in the model is different from the sign of the direct effect.

The combined effect of all mediators accounted for a significant proportion of the association between race and each of the outcomes: 41.1% for localized diagnosis, 85.0% for stage‐appropriate evaluation, 75.8% for stage‐appropriate treatment, 94.2% for two‐year survival, and 51.1% for optimal care (all *p* < 0.001) (Table S3). Among all mediators, Medicare‐Medicaid dual eligibility explained the greatest proportion of the effects of race on four of five outcomes: localized diagnosis stage (13.6%, *p* < 0.001), stage‐appropriate treatment (21.5%, *p* < 0.001), two‐year survival (25.0%; *p* < 0.001), and optimal care (11.7%, *p* < 0.001) (Figure 4). The association between race and stage‐appropriate evaluation was explained largely by the ICE (17.2%, *p* < 0.001). Receipt of an influenza vaccine in the 2 years prior to diagnosis had the second largest mediating effects on the associations between race and localized stage of diagnosis (9.5%, *p* < 0.001), stage‐appropriate treatment (15.3%, *p* < 0.001), and optimal care (11.4%, *p* < 0.001). Medicare‐Medicaid dual eligibility (15.4%, *p* < 0.001) and the percent of the region with a high school degree or less (17.9%, *p* < 0.001) had the second largest mediating effects for the associations between race and stage‐appropriate evaluation and race and two‐year survival, respectively.

Variables that were markers of neighborhood‐level SES accounted for the third largest proportion of the total effect of race on each outcome: percent of region with high school degree or less for localized stage of diagnosis (8.9%, *p* < 0.001), stage‐appropriate evaluation (14.9%, *p* < 0.001), and optimal care (11.4%, *p* < 0.001) and median household income for stage‐appropriate treatment (11.1%, *p* < 0.001) and two‐year survival (15.5%, *p* < 0.001). The mediating effects of frailty and having a hospitalization in the year prior to diagnosis were either not significant or accounted for the lowest proportions of the total effect of race on each outcome.

## 4 Discussion

In this population‐based study of Medicare beneficiaries diagnosed with NSCLC, we identified several markers of exposure to structural racism that mediate the association between race and cancer care. We first demonstrated that, even in contemporary practice, racial inequities exist at each phase of NSCLC care. We then highlighted several patient‐ and neighborhood‐level factors related to health status, access to health care, SES, and segregation that partly explain these inequities. At most phases, Medicare‐Medicaid dual eligibility had the largest mediating effect. Notably, however, the relative strength of each mediator varied depending on the phase of care. These results highlight the role that factors tied to structural racism play in promoting racial inequities across the spectrum of NSCLC care. Importantly, our results suggest that interventions to mitigate inequities should be adapted to the phase of care, as the drivers of inequity are not uniform along the care continuum.

Prior studies have put forth explanatory models detailing the proximate phase‐specific barriers that drive inequitable NSCLC care. Our study builds upon these models by describing the associated upstream, structural factors underlying inequity. For example, at the diagnosis and evaluation phases, equity is threatened by differences in the timely completion of specialist referrals [40, 41] as well as differential access to screening [42] and imaging technologies [2]. Timely completion of specialist referrals and regular screening require reliable access to health care services. This was reflected in our findings that, after dual eligibility, the second strongest mediator of racial inequities at the diagnosis phase was receipt of an influenza vaccine in the last 2 years, a marker of access to health care. Additionally, having a PCP visit in the last 2 years, another marker of health care access, was a significant mediator across all phases but exhibited its strongest effects at the evaluation phase. On the other end of the continuum, during survivorship, when patients are recovering from treatment [43], equity is threatened by differences in baseline comorbidity burden [11, 12, 44]. Two markers of patient health status—frailty and prior hospitalization—accounted for the greatest proportion of racial inequities at this phase. Our study, therefore, demonstrates that the pathway to inequitable NSCLC care comprises several upstream factors that have varying influences based on the specific demands presented at each phase of care.

Our description of health care access, health status, and other structural factors driving inequity in NSCLC care suggests these upstream factors are potential targets for interventions aiming to promote equity. Efforts to mitigate cancer inequities often focus on the various proximate, downstream factors. For example, low‐quality care at the diagnosis and evaluation phases has been addressed through the development of mobile cancer screening units [45] and the implementation of care navigator programs [46]. Our results suggest that these interventions can be augmented with levers of change addressing access to health care, individual SES, and other phase‐specific upstream mediators. One possible intervention is the design of cash transfer programs, initiatives where direct cash payments are made to patients to mitigate the socioeconomic burden of cancer care. Early evidence suggests that these programs improve access to preventative health services [47, 48] and health outcomes [49, 50, 51]. Bolstering the evidence base regarding the effectiveness of these and other programs targeting upstream drivers of inequity can help promote equity along all phases of NSCLC care.

Previous work has shown that Medicare‐Medicaid dual eligibility is associated with low‐quality care for lung [52, 53, 54, 55], colon [54, 55, 56, 57], breast [54, 55, 56], and gynecological cancer [54, 55, 58]. Our study expands on these findings and demonstrates that, in the case of NSCLC, dual eligibility is a prominent mediator of racial inequities across the spectrum of cancer care. Dually eligible patients are Medicare beneficiaries who also qualify for Medicaid based on having income levels below a specific threshold. This threshold varies by state but is generally set at or below 100%–138% of the federal poverty level [59]. Dual eligibility is, therefore, often used as a proxy for SES [52, 60, 61]. The role of dual eligibility in explaining racial inequities may be tied to the wide range of health‐related social needs (HRSNs) that dually eligible individuals experience. Compared to Medicare‐only beneficiaries, patients who are dually eligible for Medicaid have greater social support needs [30], more food and housing insecurity [62], and less reliable transportation [62]. HRSNs are known to influence risk factors for the development and progression of cancer and quality of care at all phases of the cancer care continuum [63, 64]. Accordingly, there has been increasing focus on screening cancer patients for HSRNs [65, 66, 67]. Our finding that dual eligibility was a prominent mediator across the spectrum of NSCLC care warrants characterization of any potential HRSNs that may be contributing to worse cancer care for NSCLC patients.

Our study should be interpreted in the context of key limitations. The Black population eligible for Medicare is a diverse group, comprising Afro‐Caribbean, Afro‐Hispanic, African American, and multiracial persons [68]. Moreover, a subset of Medicare beneficiaries may not identify with any racial groups, or their identities may be grounded in genetic ancestry testing, information we could not obtain from SEER‐Medicare. Because the lack of granular race information in SEER‐Medicare largely precludes a nuanced understanding of the mechanisms underlying inequity in NSCLC care for these specific sub‐populations, we elected to focus on as homogenous a population as possible by limiting the sample to patients of non‐Hispanic ethnicity. Ultimately, the most accurate portrayal of the identities of patients would come from patient‐reported data. Future studies should consider using patient‐reported data to more accurately capture race‐related identities.

Additionally, the underlying assumptions of our conceptualized mediation models may be undermined by the presence of unmeasured confounders and mediators. Therefore, the reported effects are best viewed as associative rather than definitive causal effects, especially given the observational nature of our data. An example of a potentially missed mediator is a history of cigarette smoking. Cigarette smoking is a strong risk factor for NSCLC diagnosis and is an important part of lung cancer screening guidelines. Importantly, this negative health behavior has been plagued by forces of structural racism, including targeted consumer advertising and marketing in Black neighborhoods [69, 70, 71]. In this regard, cigarette smoking may have been a significant mediator of racial inequities in NSCLC care, particularly at the diagnosis phase of care. Finally, operationalizing appropriate care with dichotomized “yes/no” outcomes may not accurately measure the quality of care. Nonetheless, our study, which used a large national cohort of patients and examined care comprehensively at each phase of the NSCLC continuum, provides valid insights into the quality of care and the inequities that underlie it.

## 5 Conclusions

This study found that several markers representing exposure to structural racism account for the worse cancer care that Black patients experience along the continuum of NSCLC care. Importantly, the factors that mediate these racial inequities vary in relative strength along the NSCLC care continuum, suggesting that interventions to mitigate inequities should be tailored to the specific phase of care. For most phases, Medicare‐Medicaid dual eligibility, a marker of individual SES, had the strongest mediating effects. There is also an opportunity to address inequities along each phase of care stemming from patient access to health care, neighborhood socioeconomic disadvantage, and residential and economic segregation.

## Author Contributions

**Safraz A. Hamid:** conceptualization (equal), formal analysis (lead), investigation (lead), methodology (lead), writing – original draft (lead), writing – review and editing (lead). **Do H. Lee:** conceptualization (equal), formal analysis (supporting), investigation (supporting), methodology (supporting), writing – review and editing (supporting). **Jeph Herrin:** formal analysis (supporting), investigation (supporting), methodology (supporting), writing – review and editing (supporting). **James B. Yu:** investigation (supporting), writing – review and editing (supporting). **Craig E. Pollack:** investigation (supporting), writing – review and editing (supporting). **Lorraine T. Dean:** investigation (supporting), writing – review and editing (supporting). **Jacquelyne J. Gaddy:** investigation (supporting), writing – review and editing (supporting). **Carol R. Oladele:** investigation (supporting), writing – review and editing (supporting). **Shelli L. Feder:** investigation (supporting), writing – review and editing (supporting). **Maureen E. Canavan:** investigation (supporting), writing – review and editing (supporting). **Marcella Nunez‐Smith:** writing – review and editing (supporting). **Pamela R. Soulos:** data curation (lead), formal analysis (supporting), investigation (supporting), methodology (supporting), project administration (supporting), resources (supporting), supervision (supporting), writing – review and editing (supporting). **Cary P. Gross:** conceptualization (lead), data curation (supporting), formal analysis (supporting), funding acquisition (lead), investigation (supporting), methodology (supporting), project administration (lead), resources (supporting), supervision (lead), writing – review and editing (supporting).

## Ethics Statement

The Yale Human Investigation Committee determined that this study did not constitute human participants research and was exempt from full IRB review.

## Conflicts of Interest

Jeph Herrin: Dr. Herrin receives funding from multiple institutes of the National Institutes of Health, from the Patient‐Centered Outcomes Research Institute, the American Heart Association, and the Agency for Healthcare Research and Quality for research projects; from the Centers for Medicare and Medicaid Services for development of quality measures; and from Pfizer. James B. Yu: Dr. Yu reports speaking fees and a research grant from Pfizer/Myovant, consulting fees from Boston Scientific, and stock in Modifi Bio. Craig E. Pollock: Dr. Pollack reports stock ownership in Gilead Pharmaceuticals outside the submitted work; he is a board member of the American Association of Service Coordinators; Johns Hopkins contracted with the US Department of Housing and Urban Development for him to assist the agency on housing and health issues from September 2019 to July 2022. Cary P. Gross: Dr. Gross has received research funding from the NCCN Foundation (with funds provided to NCCN by Astra‐Zeneca) and Genentech, as well as funding from Johnson and Johnson to help devise and implement new approaches to sharing clinical trial data. The other authors declare no conflicts of interest.

## Supporting information


Table S1.

Table S2.

Table S3.

Figure S1.

## Data Availability

The data underlying this article are available in Surveillance, Epidemiology, and End Results (SEER)‐Medicare at https://healthcaredelivery.cancer.gov/seermedicare/. The data we used from SEER‐Medicare cannot be shared according to the terms of our Data Use Agreement (DUA).
