# Supplementary material for: Mediators of Racial Inequities in Non‐Small Cell Lung Cancer Care
Source: Cancer Med. 2025 Mar 7;14(5):e70757. doi: 10.1002/cam4.70757 (PMC11886416; doi:10.1002/cam4.70757)
Supplement: Supplementary file 1 — Table S1. [file CAM4-14-e70757-s003.docx]

| **eTable S1. Stage-appropriate evaluation based on NCCN guidelines.** | | |
| --- | --- | --- |
|  | 2013-2016 | 2017 v3 |
| Stage IA | PFT (if not previously done)  Bronchoscopy  Pathologic mediastinal lymph node evaluation (category 2B; mediastinoscopy, mediastinotomy, EBUS, EUS, and CT-guided biopsy.)  PET/CT scan (if not previously done) | PFT (if not previously done)  Bronchoscopy  FDG PET/CT scan (if not previously done) |
| Stage IB | PFT (if not previously done)  Bronchoscopy  Pathologic mediastinal lymph node evaluation (mediastinoscopy, mediastinotomy, EBUS, EUS, and CT-guided biopsy.)  PET/CT scan (if not previously done)  Brain MRI | PFT (if not previously done)  Bronchoscopy  Pathologic mediastinal lymph node evaluation (mediastinoscopy, mediastinotomy, EBUS, EUS, and CT-guided biopsy.)  FDG PET/CT scan (if not previously done) |
| Stage II  Stage IIIA | PFT (if not previously done)  Bronchoscopy  Pathologic mediastinal lymph node evaluation (mediastinoscopy, mediastinotomy, EBUS, EUS, and CT-guided biopsy.)  PET/CT scan (if not previously done)  Brain MRI | |
| Stage IIIB (T1-3, N3) | PFT (if not previously done)  PET/CT scan (if not previously done)  Brain MRI  Pathologic confirmation (mediastinoscopy, supraclavicular lymph node biopsy; thoracoscopy, needle biopsy, mediastinotomy, EUS/EBUS biopsy) | |
| Stage IIIB (not T1-3/N3) | PET/CT scan (if not previously done)  Brain MRI  Pathologic confirmation (mediastinoscopy, supraclavicular lymph node biopsy; thoracoscopy, needle biopsy, mediastinotomy, EUS/EBUS biopsy) | |
| Stage IIINOS | PET/CT scan (if not previously done)  Brain MRI  Pathologic mediastinal lymph node evaluation (mediastinoscopy, mediastinotomy, EBUS, EUS, and CT-guided biopsy.) OR Pathologic confirmation (mediastinoscopy, supraclavicular lymph node biopsy; thoracoscopy, needle biopsy, mediastinotomy, EUS/EBUS biopsy) | |
| Stage IV  Adenocarcinoma, large cell, NSCLC NOS (i.e. non-squamous) | Any molecular testing | |
