# Supplementary material for: Mediators of Racial Inequities in Non‐Small Cell Lung Cancer Care
Source: Cancer Med. 2025 Mar 7;14(5):e70757. doi: 10.1002/cam4.70757 (PMC11886416; doi:10.1002/cam4.70757)
Supplement: Supplementary file 2 — Table S2. [file CAM4-14-e70757-s002.docx]

| **eTable S2**. Stage-appropriate treatment based on NCCN guidelines. | |
| --- | --- |
|  | 2013-2017 |
| Stage I | Surgery or RT |
| Stage IIA | Surgery or RT |
| Stage IIB (T3, N0) | If tumor size >7 cm: surgery or RT |
| Stage IIB (T3 invasion, N0) | If tumor size  ≤7 cm: surgery or chemoRT |
| Stage IIB (all others) | Surgery or RT |
| Stage IINOS | Surgery, RT, or chemoRT (any stage II treatment) |
| Stage IIIA (T1–3, N2) | Chemo+surgery or chemoRT |
| Stage IIIA (T3, N1; T4, N0-1) | Surgery or chemoRT |
| Stage IIIB | ChemoRT |
| Stage IIINOS | Surgery, chemo, or chemoRT (any stage III treatment) |
| All Stage IV | Surgery, RT, or chemo |
