# Supplementary material for: Mediators of Racial Inequities in Non‐Small Cell Lung Cancer Care
Source: Cancer Med. 2025 Mar 7;14(5):e70757. doi: 10.1002/cam4.70757 (PMC11886416; doi:10.1002/cam4.70757)
Supplement: Supplementary file 3 — Table S3. [file CAM4-14-e70757-s001.docx]

| **eTable S3. Relative Indirect Effects and 95% Confidence Intervals** | | | | | |
| --- | --- | --- | --- | --- | --- |
|  | **Localized Stage at Diagnosis** | **Evaluation** | **Treatment** | **Two-Year Survival** | **Optimal Care** |
| **Mediators** | | | | | |
| **Total Indirect Effect^a^** | 41.1 (32.3, 52.6) | 85.0 (68.2, 105.5) | 75.8 (66.8, 86.4) | 94.2 (79.8, 1.14) | 51.1 (39.7, 63.8) |
| **Health Care Access** |  |  |  |  |  |
| Flu Shot in the Last 2 Years | 9.5 (6.8 , 12.8) | 13.3 (10.0, 17.3) | 15.3 (12.9, 17.6) | 12.2 (9.6, 15.7) | 11.4 (8.3, 14.8) |
| PCP Visit in the Last 2 Years | 3.6 (2.4, 5.1) | 12.5 (9.2, 15.9) | 11.2 (9.2, 13.2) | 7.6 (5.8, 9.9) | 9.0 (6.8, 12.0) |
| **Health Status** | | | | | |
| Prior Hospitalizations in the Last Year | − | 1.1 (0.5, 1.8) | 1.4 (0.8, 2.0) | 1.5 (0.9, 2.3) | − |
| Frail | − | 2.8 (1.7, 4.2) | 3.1 (2.0, 4.3) | 4.3 (2.8, 5.9) | − |
| **Patient- and Neighborhood-level SES Factors** | | | | | |
| Dual Eligibility for Medicaid | 13.6 (9.5, 17.9) | 15.4 (10.8, 20.2) | 21.5 (18.2, 25.1) | 25.0 (20.0, 29.5) | 11.7 (7.6, 16.0) |
| % of Region with High School Degree or Less | 8.9 (4.5, 12.9) | 14.9 (10.4, 20.6) | 5.8 (2.6, 8.9) | 17.9 (12.9, 24.5) | 11.4 (8.1, 14.5) |
| % of Region Living in Poverty | − | − | 2.3 (0, 6.0) | − | − |
| Median Household Income | 3.5 (0, 8.8%) | 8.0 (3.7, 13.5) | 12.1 (8.7, 16.1) | 15.5 (10.1, 21.4) | − |
| **Segregation** | | | | | |
| ICE Index | 4.6 (0.2, 9.5) | 17.2 (10.9, 24.3) | 3.0 (0, 7.1) | 10.4 (5.4, 15.4) | 8.7 (4.1, 14.2) |
| − Indicates that relative indirect effects were either not statistically significant or demonstrated inconsistent mediation^77^  ^a^Total indirect effect also accounts for inconsistent mediation.  PCP = primary care physician; SES = socioeconomic status; ICE = Index of Concentration at the Extremes | | | | | |
