# Supplementary material for: Mediators of Racial Inequities in Non‐Small Cell Lung Cancer Care
Source: Cancer Med. 2025 Mar 7;14(5):e70757. doi: 10.1002/cam4.70757 (PMC11886416; doi:10.1002/cam4.70757)
Supplement: Supplementary file 4 — Figure S1. [file CAM4-14-e70757-s004.docx]

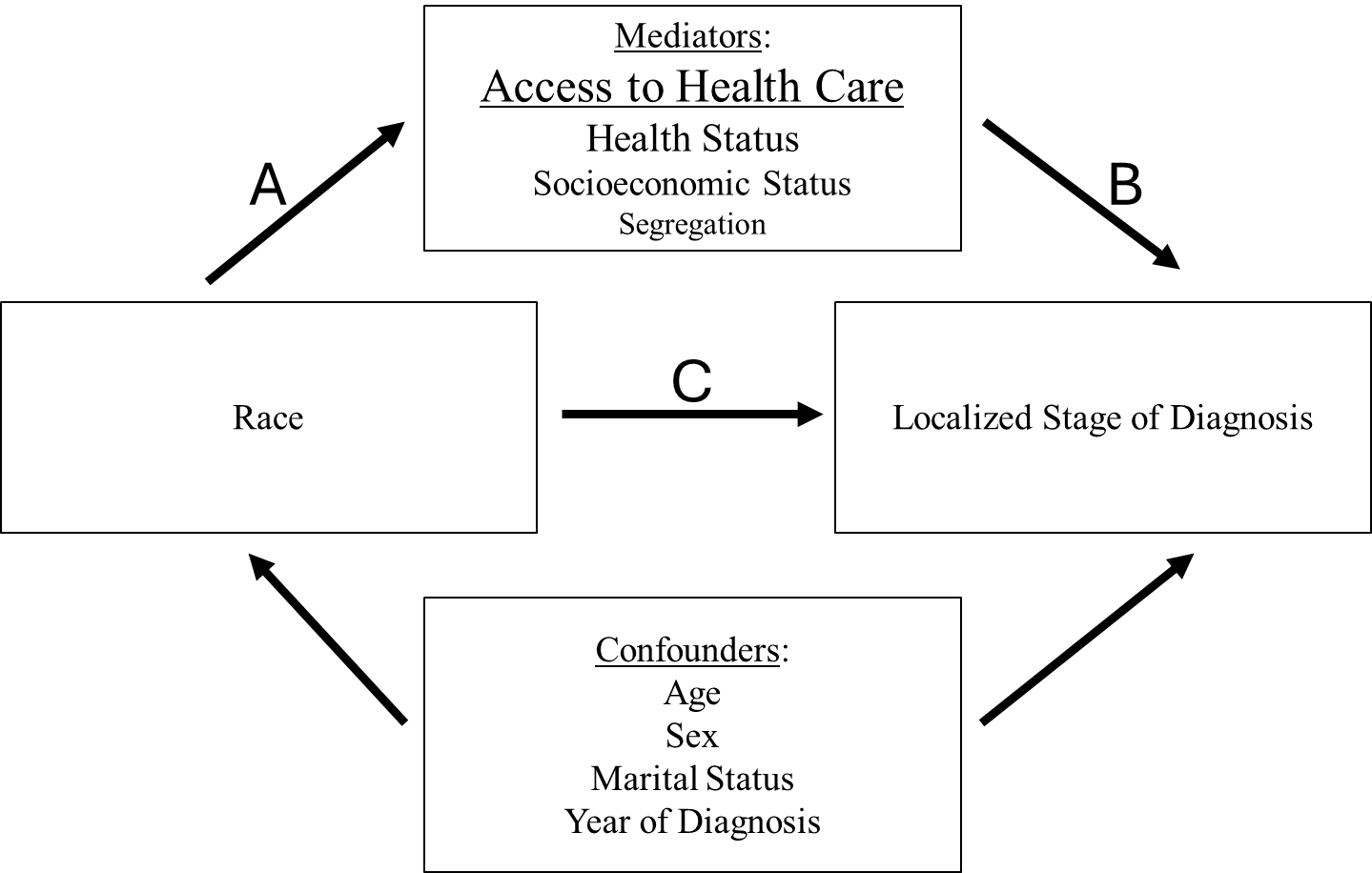


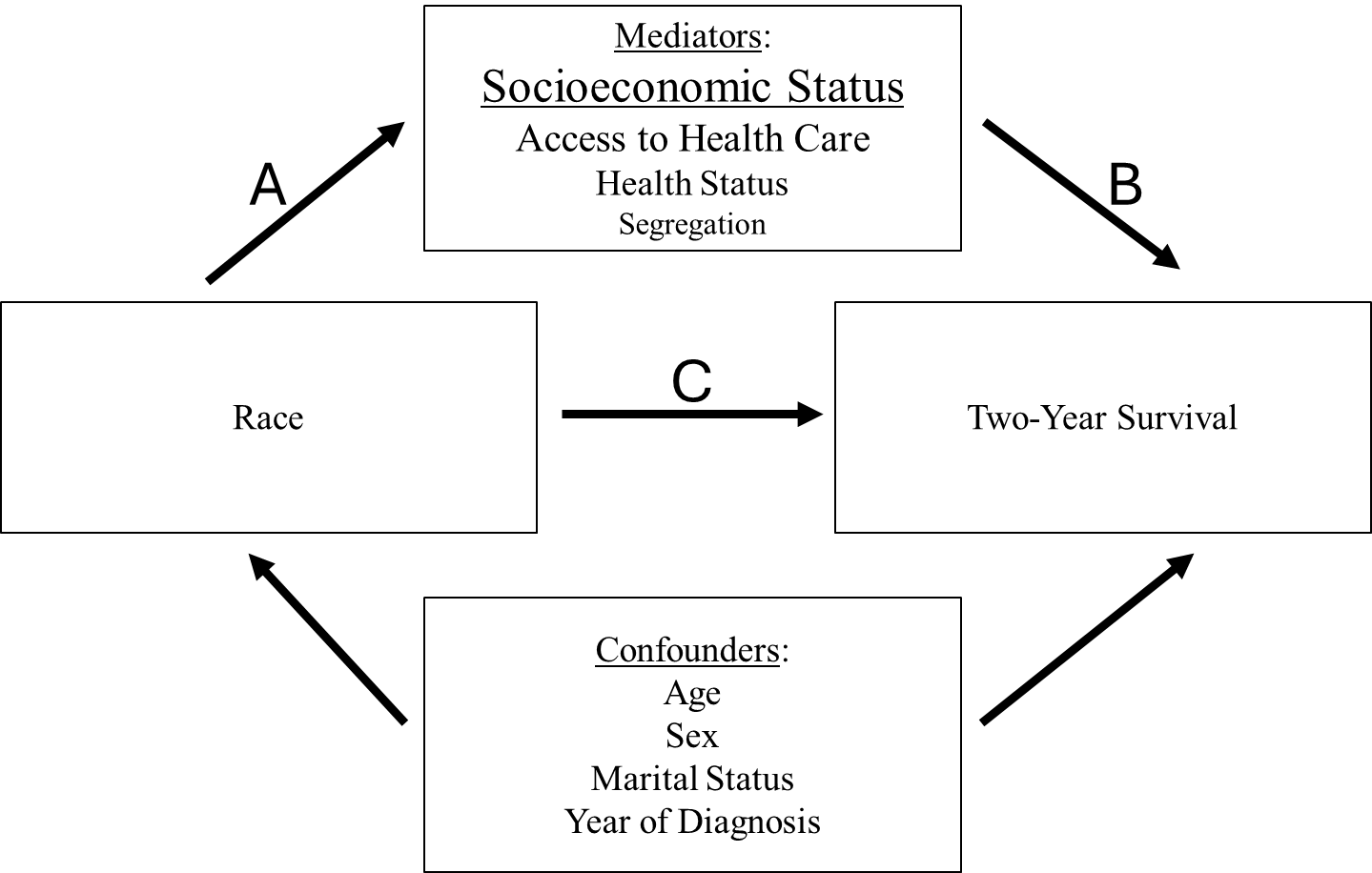


**Supplementary Figure 1.** Conceptual models for two outcomes—localized diagnosis stage and two-year survival—illustrating our hypothesis that the relative strength of mediators may differ based on the phase of care. In these models, stronger hypothesized mediation effects are depicted by a larger font of the mediator.
